# GSK3-Driven Modulation of Inflammation and Tissue Integrity in the Animal Model

**DOI:** 10.3390/ijms25158263

**Published:** 2024-07-29

**Authors:** Friederike Kühl, Korbinian Brand, Ralf Lichtinghagen, René Huber

**Affiliations:** Institute of Clinical Chemistry and Laboratory Medicine, Hannover Medical School, 30625 Hannover, Germany; kuehl.friederike@mh-hannover.de (F.K.); brand.korbinian@mh-hannover.de (K.B.); lichtinghagen.ralf@mh-hannover.de (R.L.)

**Keywords:** GSK3α, GSK3β, serine/threonine phosphorylation, inflammation, inflammatory diseases, animal models

## Abstract

Nowadays, GSK3 is accepted as an enzyme strongly involved in the regulation of inflammation by balancing the pro- and anti-inflammatory responses of cells and organisms, thus influencing the initiation, progression, and resolution of inflammatory processes at multiple levels. Disturbances within its broad functional scope, either intrinsically or extrinsically induced, harbor the risk of profound disruptions to the regular course of the immune response, including the formation of severe inflammation-related diseases. Therefore, this review aims at summarizing and contextualizing the current knowledge derived from animal models to further shape our understanding of GSK3α and β and their roles in the inflammatory process and the occurrence of tissue/organ damage. Following a short recapitulation of structure, function, and regulation of GSK3, we will focus on the lessons learned from GSK3α/β knock-out and knock-in/overexpression models, both conventional and conditional, as well as a variety of (predominantly rodent) disease models reflecting defined pathologic conditions with a significant proportion of inflammation and inflammation-related tissue injury. In summary, the literature suggests that GSK3 acts as a crucial switch driving pro-inflammatory and destructive processes and thus contributes significantly to the pathogenesis of inflammation-associated diseases.

## 1. Introduction

The paralogs of glycogen synthase kinase (GSK) 3, i.e., GSK3α and GSK3β, contribute to the regulation, modulation, and orchestration of a variety of molecular processes with implications for all physiological features of cells and organisms [1]. These include intracellular signaling, energy metabolism, proliferation, differentiation, cell death, adhesion, and migration thus steering, for instance, embryonic development, aging, tumor suppression, the immune response, as well as tissue development, remodeling, and function. Mechanistically, GSK3 is involved in signaling cascades regulating various (post-)transcriptional and (post-)translational effects [2] and influences the activity, localization, and stability of GSK3 substrates [3]. Consistently, GSK3 also plays a role under pathophysiological conditions including the acute immune response against infections with bacteria, viruses, and parasites [4] as well as the development and progression of chronic inflammatory diseases [5] such as rheumatoid arthritis [6], colitis [7], and hepatitis [8]. Moreover, GSK3 has been recognized in diseases with a significant inflammatory component, e.g., diabetes [9] or neurodegenerative disorders such as Alzheimer’s (AD), Parkinson’s (PD), and Huntingdon’s disease [10]. The same applies to multiple forms of cancer [11], which are also associated with inflammatory events [12].

Structure, function, and regulation of GSK3 have been extensively reviewed by our group [2] and others (e.g., [3,13,14,15]). Therefore, this review will only provide a short summary of these aspects. The focus, however, will be the presentation and discussion of findings derived from various animal models illuminating the pro- and anti-inflammatory as well as tissue protective or destructive qualities of GSK3. This includes GSK3 knock-out (KO) and knock-in (KI)/overexpression models as well as models designed for studying diseases which are significantly driven by inflammatory events.

## 2. GSK3—A Condensed Overview on Structure, Function, and Regulation

**Figure 1:**
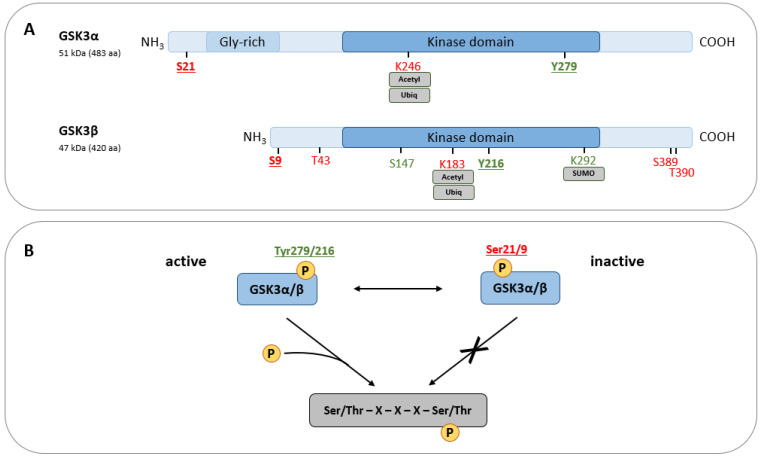
(**A**) Structural and functional domains of GSK3 paralogs α and β. Regulatory sites are indicated depending on their activating (green) or inhibiting (red) function. Unless otherwise indicated, the respective residues are post-translationally modified via phosphorylation. (**B**) GSK3 activity is predominantly regulated through phosphorylation. Ser21/9 are the major inhibitory GSK3 phosphorylation sites, whereas residues Tyr279/216 determine its activation. In the presence of a C-terminal priming phosphorylation mediated by other kinases, active GSK3 phosphorylates target proteins at the consensus sequence Ser/Thr–X–X–X–Ser/Thr.

GSK3 is a monomeric serine/threonine kinase [16], initially identified to be involved in glycogen metabolism by targeting glycogen synthase (GS) [17]. Later, sequence analyses revealed that the GSK3 family comprises two highly similar paralogs, namely the 51 kDa variant GSK3α and the slightly shorter GSK3β (47 kDa; Figure 1A), which are characterized by high sequence similarity (approx. 85% in total and 98% in the catalytic region) [18,19] and widely conserved between different species [20]. Both paralogs contain a crucial negative regulatory N-terminal domain, the kinase domain (mediating ATP binding and enzymatic function), and a C-terminal domain also possessing several (though less influential) negative regulatory sites. Despite their remarkable similarity and some overlapping functions (e.g., in Wnt/β-catenin signaling [21]), both enzymes are differentially expressed (including tissue-specific expression patterns [22]), target different sets of substrates, and regulate divergent cellular functions [2]. Moreover, the loss of one paralog can neither be fully compensated by nor induces the expression or activity of the remaining paralog [21,23].

To date, approx. 100 proteins are reasonably considered as GSK3 substrates (including prominent representatives like β-catenin, tau protein, or protein phosphatase 1) [24] and over 400 more have been proposed, but need validation [1]. Following priming phosphorylation of the C-terminal Ser/Thr residue by other kinases (e.g., casein kinase II), GSK3 phosphorylates proteins at the consensus sequence Ser/Thr-X-X-X-Ser/Thr (Figure 1B). In the presence of several consecutive consensus sequences within a specific substrate, however, GSK3 is able to provide its own priming phosphorylation once the initial priming is established [25].

Interestingly, GSK3 is characterized by constitutively high basal activity which can be negatively modified by post-translational modifications resulting in a fast suppression of enzymatic activity [14,23]. A keystone of basal GSK3 activity is activating phosphorylation at Tyr279 (GSK3α) and Tyr216 (GSK3β), respectively, which facilitates proper folding of the catalytic domain [24] and increases substrate accessibility [26], catalytic turnover [27], and GSK3 protein stability [28]. Both residues are already phosphorylated during protein translation of GSK3 [29], either by certain kinases (e.g., src or MAPK family members) [30] or autophosphorylation [28], and appear to be relatively stable [28]. Nonetheless, under certain conditions, dynamic regulation of Tyr279/216 phosphorylation has been described [31]. The most prominent regulatory sites, however, are Ser21 (GSK3α) and Ser9 (GSK3β) since their phosphorylation by various kinases (e.g., PKC, p38, S6K, RSK, or Akt) in response to a variety of stimuli (e.g., cytokines, growth factors, or insulin) [2] allows the N-terminus to act as a catalytic domain-blocking pseudo-substrate [32]. In consequence, dephosphorylation of Ser21/9 by protein phosphatases results in (re-)activation of GSK3 [33]. In GSK3β, additional activating (e.g., Ser147) and inhibitory phosphorylation sites (e.g., Thr43, Ser389, and Thr390) as well as additional post-translational modifications, either supporting (SUMOylation, citrullination) or mitigating (acetylation, mono-ADP-ribosylation) its function, have been described [2] (Figure 1).

## 3. KO and KI Models

Various conventional and conditional KO as well as KI models (including constitutively active variants) have been developed and used to study the impact of GSK3α and β deficiency or overexpression on animal health and development [23,34].

### 3.1. Conventional GSK3α KO

**Figure 2:**
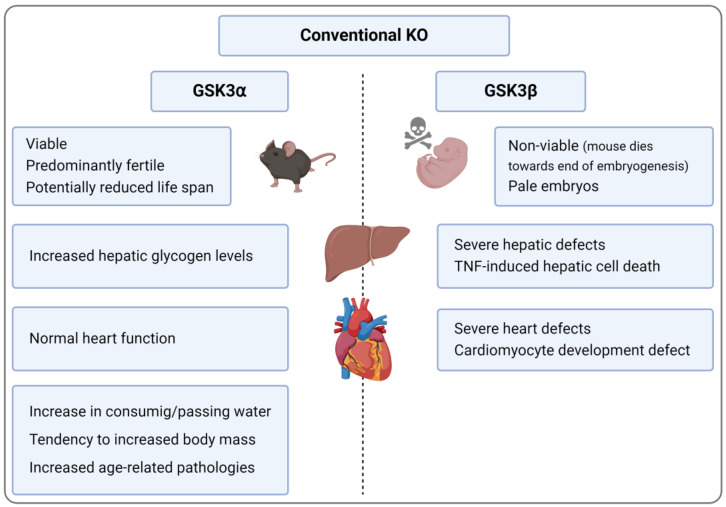
Comparison of GSK3 deficiency-related, organ-specific effects observed in GSK3α and GSK3β KO mice. Created with BioRender.com.

In general, GSK3α KO mice are relatively normal, i.e., they are both viable and fertile and exhibited a normal brain size (despite minor alterations in the distribution of tissues and cell types) [35], body weight [36], and skeletal development [37]. The main deviations were an increase in consuming and passing water [38], hepatic glycogen levels, and sensitivity towards challenges with certain stimuli, resulting, for instance, in the enhanced insulin-dependent inhibition of GSK3β [36]. The generation of a second murine GSK3α KO model yielded a somewhat different phenotype, since these mice showed a slightly reduced lifespan. They were further characterized by enhanced body mass, heavier organs, and male infertility [39]. Another group found evidence for reduced life span and increased age-related pathologies, e.g., cardiac hypertrophy, contractile dysfunction, and cardiac/skeletal muscle sarcopenia, muscle degeneration, increased senescence in liver/small intestine, earlier onset of osteoarthritis, and suppressed autophagy [40] (Figure 2). Moreover, in an atherogenesis-favoring background (i.e., low-density lipoprotein receptor (LDLR) KO mice under high-fat diet (HFD)), GSK3α deficiency led to smaller atherosclerotic lesions, increased anti-inflammatory IL-10 levels in the plasma, and attenuated hepatic steatosis [41].

### 3.2. Conditional GSK3α KO

In a cardiomyocyte-specific KO approach, loss of GSK3α turned out to preserve left ventricular function and to limit tissue remodeling following myocardial infarction surgery [42] or trans-aortic constriction surgery [43], presumably due to thicker scar formation and enhanced cardiomyocyte viability and proliferation in the affected mice [42]. GSK3α further appears to play a role in proper spermatogenesis and male fertility as shown in a murine testicular germ cell-specific GSK3α KO model [44]. Neuron-specific GSK3α KO mice showed a normal life span, neural anatomy, body and brain weight, motor activity, and fertility, but alterations in the synaptic transmissions [39].

In LDLR KO mice, myeloid-specific GSK3α deficiency impaired inflammatory M1 [45] and favored M2 macrophage polarization [46]. Furthermore, pro-inflammatory signaling (e.g., nuclear factor (NF-)κB, NLR family pyrin domain containing (NLRP) 3) and cytokine expression (e.g., TNF, IL-6) were decreased, while macrophage migration was enhanced [45]. Under atherosclerosis-inducing conditions (i.e., HFD), atherosclerotic lesion size, volume, and complexity as well as TNF and IL-6 plasma levels were reduced in this KO model, while anti-inflammatory IL-10 was more abundant [46]. An equivalent amelioration of atherosclerosis—including reduced plaque volume, necrotic core area, monocyte/macrophage recruitment, and expression of adhesion molecules and pro-inflammatory proteins, but increased plaque stability—could be observed when GSK3α was absent in macrophages, endothelial cells, or both [47,48]. Overall, neither conventional nor conditional GSK3α KO animals appear to be prone to alterations in inflammatory processes under standard conditions, but showed anti-inflammatory effects under inflammation-supporting conditions.

### 3.3. Conventinal and Conditional GSK3β KO

Conventional GSK3β KO models are not available since the respective mice die towards the end of embryogenesis [23] due to severe hepatic and cardiac defects mainly related to a TNF-dependent increase in hepatic cell death [49] and disturbances in the proper development of cardiomyocytes [50] (Figure 2). These problems could be avoided by conditional GSK3β KO models, which predominantly produce viable and utilizable animals with a distinct phenotype.

#### 3.3.1. General Aspects of Conditional GSK3β KO

Renal collecting duct-specific GSK3β KO mice, for instance, showed only mild polyuria under normal conditions but a reduced ability of urine concentration, e.g., in response to water deprivation [51]. GSK3β KO in the renal proximal tubule even proved to be protective against mortality and tubular injury due to rapid tissue regeneration as reflected by reduced apoptosis in the renal cortex and accelerated proliferation of renal proximal tubule cells in a mercury chloride-induced acute nephrotoxic injury model [52]. In humans with and experimental models of proteinuric glomerulopathy, GSK3β expression was markedly enhanced in podocytes [53]. Accordingly, in murine experimental adriamycin nephropathy [53] and oxidative glomerular injury [54], podocyte-specific GSK3β KO significantly decreased podocyte loss and injury, reduced glomerular damage, attenuated proteinuria [53,54], and diminished glomerular reactive oxygen species (ROS) production [54,55]. The respective podocytes were characterized by increased glycogen accumulation [54] as well as preserved cytoskeleton integrity and focal adhesions, reduced mitochondria dysfunction, and diminished pro-inflammatory NF-κB activation [53].

Though the proper formation of serotonin neurons and neuronal tissue was not affected, serotonin neuron-specific GSK3β KO mice showed a reduced inhibitory response towards stimulation with the serotonin 1B receptor agonist anpirtoline resulting in increased serotonin secretion, serotonergic neuron firing, and alterations in serotonin-regulated behavior [56]. Anti-apoptotic effects of GSK3β deficiency have been observed in murine GSK3β KO oligodendrocytes that are protected from caspase-dependent (but not -independent) apoptosis. Under pro-apoptotic conditions (i.e., cuprizone treatment), these mice were characterized by myelin preservation in combination with reduced glia cell activation [57]. Another study demonstrated that conditional GSK3β KO in a subset of cortical and hippocampal neurons leads to alterations in spine density and morphology, i.e., reduced amounts and formation of most spines [58].

Following HFD, mice with cardiomyocyte-specific GSK3β KO developed increased heart/lung weight and suffered from severe cardiac dysfunction and adverse ventricular remodeling [59]. Beta cell-specific GSK3β KO led to an increase in glucose tolerance and beta cell mass/proliferation [60]. In hematopoietic cell-specific GSK3β KO mice, normal hematopoiesis was observed [61], and mice with liver-specific GSK3β KO did not exhibit metabolic abnormalities or alterations in insulin signaling, though activity of total GSK3 in liver extracts was significantly reduced. In contrast, skeletal muscle-specific GSK3β KO resulted in enhanced skeletal muscle glycogen storage and clearance of blood glucose in the respective mice, an effect accompanied by normal insulin levels, but increased insulin signaling (i.e., GSK3α inhibition and GS activation) [62]. During myogenesis, enhanced mitochondrial metabolism and respiration have also been observed in this model [63]. Together, these reports suggest that under most conditions, cell type-/tissue-specific GSK3β deficiency is able to mediate tissue protection and functional preservation by enhancing tissue regeneration, while reducing apoptosis and oxidative stress.

#### 3.3.2. Inflammation-Associated Aspects of Conditional GSK3β KO

In a model of ischemia–reperfusion (IR) injury applied to mice with GSK3β KO in the myeloid lineage, reduced expression of pro- (e.g., TNF, IL-6) and increased expression of anti-inflammatory cytokines (IL-10) could be observed [64,65] in combination with reduced neutrophil infiltration/activation, enhanced protection of liver tissue [64], increased restoration of liver homeostasis, and multiple molecular and cellular signs for augmented resolution of inflammation [65]. In contrast, in LDLR KO mice, myeloid cell-specific GSK3β deficiency enhanced M1 macrophage polarization, pro-inflammatory signaling (e.g., NF-κB, NLRP3), and cytokine expression (e.g., IL-1α, β), while decreasing macrophage migration [45]. Following HFD-induced manifestation of atherosclerosis, however, no significant differences among GSK3β wildtype (WT) and KO mice could be observed in these animals [47,48]. When mice with renal tubule-specific GSK3β KO were challenged with mercury chloride [52] or subjected to renal IR [66] to induce acute nephrotoxic/kidney injury, they showed increased expression of pro-proliferative factors (e.g., β-catenin, cyclin D1) [66], accelerated renal tubule cell proliferation, reduced tubular injury [52,66], and decreased apoptosis in the renal cortex [52]. Equivalent anti-apoptotic effects have also been described in GSK3β-negative oligodendrocytes [57]. In models of oxidative glomerular injury [54] and adriamycin nephropathy [53], mice with podocyte-specific GSK3β KO were characterized by decreased podocyte loss, glomerular damage, and ROS formation. Moreover, in mice with hematopoietic cell-specific GSK3β deficiency and heterozygous GSK3β^+/−^ mice, signs of inflammation were significantly reduced in a peritonitis model [67]. These data suggest that GSK3β acts as a central regulator of immune cell functions and of cellular processes associated with inflammatory events. Its precise role, however, appears to be strictly context-specific, yielding in part conflicting results.

### 3.4. GSK3α/β Double KO (DKO)

GSK3α/β DKO led to impaired differentiation processes (e.g., the inability to form embryoid bodies), unregulated Wnt signaling, and massive upregulation of β–Catenin levels in murine embryonic stem cells [21]. In a neural progenitor-specific DKO mouse model, strong disturbances during neurogenesis were observed as reflected by massively enhanced proliferation of these progenitors. In contrast, the formation of intermediate progenitors as well as postmitotic neurons was significantly reduced, presumably due to an activation of several signaling pathways (β catenin, Jun, Notch, and Myc) [68]. A comparable DKO in astrocytes resulted in larger brain formation and increased numbers/size of astrocytes [69]. Postnatal induction of cartilage-specific DKO significantly impaired skeletal development, body weight, size, and breathing, accelerated growth plate remodeling, and enhanced chondrocyte apoptosis at a young age resulting in premature death [70]. LDLR KO mice with myeloid GSK3α/β DKO were similar to GSK3β KO mice (see Section 3.3), i.e., they showed increased M1 polarization, pro-inflammatory signaling, and cytokine expression, but less macrophage migration [45]. Thus, in contrast to paralog-specific KO, which predominantly resulted in mild alterations, the loss of both GSK3α and β yielded severe deleterious effects for the affected tissues/organs. This indicates that in many cell types, the presence of at least one paralog is necessary for a sound execution of basic cellular processes such as proliferation and differentiation.

### 3.5. KI Models

A variety of KI models have also been established [23,34]. In general, WT GSK3α/β single and double (D)KI mice are viable, fertile, normally developed, and not diabetic [71]. Overexpression of the constitutively active variants GSK3α-S21A and/or GSK3β-S9A results in increased glucose uptake by murine muscle cells [71] and impaired dendritic growth [72]. Phosphorylation-resistant GSK3 DKI further reduced thrombin-dependent, but increased collagen-dependent, platelet activation, and single KI approaches revealed that the first effect depended on GSK3β-S9A, while the second was due to GSK3α-S21A activity [73]. In other GSK3-S21/9A DKI models, mice were protected from developing metabolic syndrome (presumably due to enhanced adiponectin production) [74] or exhibited a moderate increase in apoptosis at the intestinal villi tips, while intestinal tissue architecture, cell proliferation, and differentiation remained normal [75].

Mice overexpressing WT GSK3β in skeletal muscle were characterized by increased body weight, fat mass, cholesterol levels, and hepatic glycogen levels, but reduced GS activity and glycogen levels in the muscle. In addition, male mice showed glucose intolerance and increased levels of insulin, non-esterified fatty acids, and triglycerides [76]. GSK3β (or GSK3β-S9A [77]) overexpression in cortical and hippocampal neurons results in AD-like neurodegeneration as indicated by increased phosphorylation and somatodendritic localization of tau proteins in hippocampal neurons [78]. These neurons were also characterized by altered morphology, frequent detachment from surrounding neuropil, and enhanced apoptosis. Increased reactive astrocytosis and microgliosis were also observed [78]. In consequence, these mice had considerable learning deficits and displayed behavioral problems [79]. These effects could be reversed by suppression of GSK3β overexpression [80]. Elevated levels of GSK3β-S9A in the brain and spinal cord [77] led to reduced volume and weight of these organs due to increased neuronal density and reduced neuronal size [81]. Over time, GSK3β-S9A overexpression in pancreatic beta cells leads to impaired glucose tolerance and reduced beta cell mass in the affected mice (due to impaired proliferation) [82]. Thus, overexpression of both WT and constitutively active GSK3 variants can lead to perceivable disturbances of physiological processes. However, as in the case of KO models, GSK3 KI resulted in tissue-specific effects, since certain cell types such as neurons are particularly affected, while others appear to be more tolerant against GSK3 overexpression or activation.

## 4. Models of Inflammatory/Inflammation-Associated Diseases

For the analysis of inflammation-related pathologies, a variety of different (mostly rodent) disease models has been developed. Regulation and activity of GSK3 as well as its impact on inflammatory processes and the associated tissue injuries have been studied under various conditions in a large part of these models. In the following, we will provide a representative survey of the available literature.

### 4.1. Arthritic Diseases

**Figure 3:**
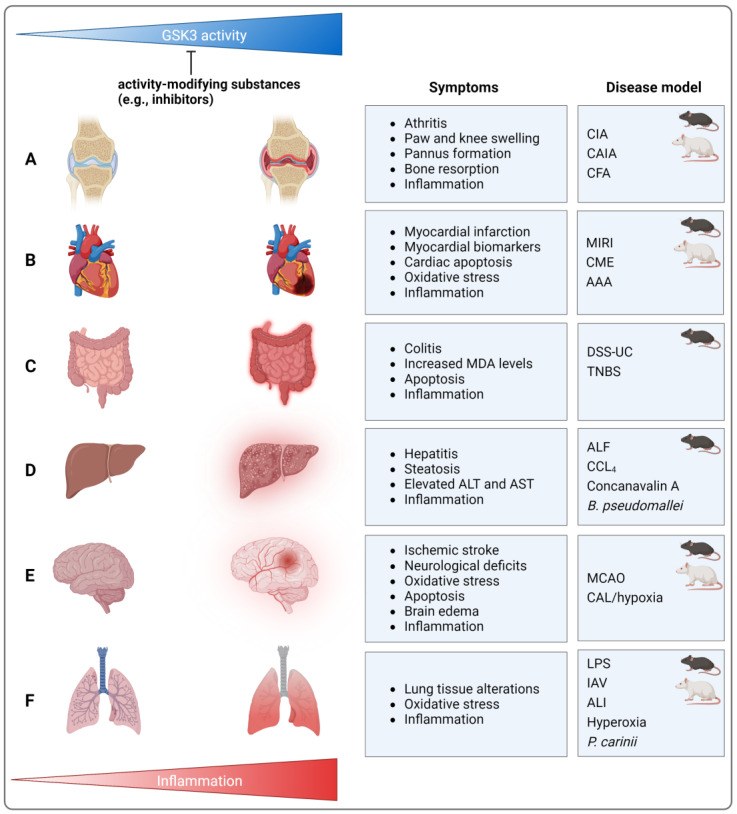
GSK3 activity is increased in various inflamed tissues associated with arthritis (**A**), vascular diseases (**B**), colitis (**C**), hepatitis (**D**), pulmonary diseases (**E**), and ischemic stroke (**F**). Among the symptoms observed in rodent models (mouse and rat), multiple signs of inflammation, oxidative stress, and tissue injury can be found. CIA, collagen-induced arthritis; CFA, complete Freund’s adjuvant; CAIA, collagen antibody-induced arthritis; MIRI, myocardial ischemia–reperfusion injury; CME, coronary micro-embolization; AAA, abdominal aortic aneurysm; MDA, malondialdehyde; DSS-UC, dextran sulfate sodium-induced ulcerative colitis; TNBS, trinitrobenzene sulfonic acid; ALT, alanine transaminase; AST, aspartate transaminase; ALF, acute liver failure; CCL4, carbon tetrachloride; MCAO, middle cerebral artery occlusion; CAL, carotid artery ligation; LPS, lipopolysaccharide; IAV, influenza A virus; ALI, acute lung injury. Created with BioRender.com.

Murine collagen-induced arthritis (CIA) is a prominent mouse model for human rheumatoid arthritis [83]. Treatment with different GSK3 inhibitors (thiadiazolidinone-8 (TDZD-8, LiCl) (for an overview of GSK3 inhibitors, please see Table 1 at the end of Section 4.7) resulted in reduced paw swelling, pannus formation, bone resorption, pro-inflammatory cytokine expression (TNF, IL-1β, IL-6, and IFN-γ), and decreased numbers of infiltrating macrophages and T-cells [84] (Figure 3). Equivalently beneficial effects of GSK3 inhibition could be observed in rat CIA using TDZD-8 [85], when murine CIA was intensified by lipopolysaccharide (LPS) injection (LiCl, valproic acid) [86], or when murine collagen antibody-induced arthritis was supplemented by toll-like receptor 1/2-activating Pam3CSK4 administration (LiCl) [67]. Appropriately, it has been described that the anti-inflammatory and destructive effects of natural compounds such as wilforine or anemoside B4 are associated with reduced GSK3β expression [87] or activity [88].

Symptoms of arthritic diseases can also be induced by injection of complete Freund’s adjuvant (CFA) [89]. Following intra-articular application of CFA, mice exhibited knee swelling, impaired locomotion, mitochondrial dysfunction, neuroinflammation, and immune cell infiltration in the spinal cord, as well as upregulation of pro-inflammatory signaling pathways (e.g., NF-κB, NLRP3) and cytokine production (IL-1β). These findings were associated with increased GSK3β activity (i.e., reduced Ser9 phosphorylation) and could be ameliorated by GSK3 inhibition (TDZD-8) [90], resembling data obtained recently in a murine CIA model [88]. Therefore, GSK3 inhibition is still regarded as a promising anti-rheumatic approach [6] (Figure 3).

These reports argue for a significant involvement of enzymatically active GSK3(β) in driving severe inflammation, either locally or systemically, while its inactivation appears to have alleviating effects. In a rat adjuvant arthritis model, however, opposing observations have been made. Here, arthritic characteristics (e.g., paw swelling, synovial hyperplasia, immune cell infiltration, and pro-inflammatory cytokine levels) occurred in the presence of reduced GSK3β protein levels combined with elevated Ser9 phosphorylation, while therapeutic application of the natural product shikonin enhanced GSK3β activity [91]. An equivalent reduction of GSK3β mRNA and protein levels has been reported in an inflammatory papain-induced knee osteoarthritis rat model. Following treatment with the small anti-inflammatory molecule iguratimod, GSK3β upregulation was accompanied by mitigation of pro-inflammatory (TNF, IL-6) and destructive features (matrix-metalloproteinase (MMP-)13, histopathological changes) [92].

### 4.2. Cardiovascular Diseases

The literature attending to the relation of GSK3 and cardiovascular diseases reflects well the respective connection in arthritides. Myocardial ischemia–reperfusion injury- (MIRI-)associated damage in rats—as represented by infarct size, increased myocardial biomarkers (e.g., creatine kinase MB (CK-MB), lactate dehydrogenase (LDH)), inflammatory cytokines (TNF, IL-6), oxidative stress [93,94], and cardiac apoptosis [94]—was reduced by GSK3β-Ser9 phosphorylation-inducing conditions (Figure 3). The latter comprise, for instance, copper nanoparticles and/or exercise [93] as well as TNF-induced protein 1 knock-down (KD) [94]. Correspondingly, an alternative rat model indicated that MIRI-induced acute lung injury (ALI) was associated with GSK3β activation (i.e., decreased p-GSK3β-Ser9 levels), while the beneficial effects of ischemic post-conditioning were associated with restored GSK3β-Ser9 phosphorylation [95]. In a comparable murine model, infarct size, local cytokine levels (IL-6, keratinocyte chemoattractant (KC)), and numbers of circulating neutrophils were also reduced when inhibitory GSK3α and β phosphorylation was elevated following the application of leukotriene B4 receptor 1 antagonist LSN2792613 [96]. Furthermore, the establishment of coronary microembolization (CME; via the injection of polyethylene microspheres into the left ventricle) in rats resulted in considerable myocardial damage, increased serum levels of myocardial injury biomarkers (CK-MB, LDH, cardiac troponin I), and cardiomyocyte apoptosis (Figure 3). In the cardiac tissues of the respective CME rats, levels of phosphorylated (p-)GSK3β-Ser9 were reduced, an effect that was abolished when the rats were pretreated with resveratrol, a natural component alleviating CME-induced damages [97].

In mice, in which neuro- and cardiodegenerative Friedreich ataxia was induced by conditional cardiac frataxin KO, fatal cardiomyopathy (cardiac hypertrophy, interstitial fibrosis, and myofibrillar disarray) and enhanced cardiac oxidative stress were detected. This was connected to increased GSK3β activity (reduced Ser9 and enhanced Tyr216 phosphorylation) and decreased expression and DNA binding of nuclear factor erythroid 2-related factor 2 (Nrf2) [98], a transcription factor crucially regulating the anti-oxidative stress response [99]. Vice versa, angiotensin II-induced cardiac hypertrophy, oxidative stress, and inflammation could be prevented by GSK3β inhibition and Nrf2 activation in response to sulforaphane [100]. Using an example of vascular disease, i.e., in rats developing abdominal aortic aneurysm (AAA) due to periaortic application of CaCl_2_, it could be shown that p-GSK3β-Ser9 levels were strongly reduced in the aortic wall. Correspondingly, GSK3 inhibition by LiCl prevented AAA by reducing the production of proteases (MMP-2 and -9), ROS, and cytokines/chemokines (TNF, monocyte chemoattractant protein (MCP-)1) as well as the infiltration of inflammatory cells [101] (Figure 3).

For other rodent models, contradictory effects have been reported. In the MIRI-induced ALI model mentioned above, for instance, GSK3 inhibition by LiCl had no positive influence on MIRI. More important, LiCl even foiled the beneficial effects of ischemic post-conditioning, though the latter restored the inhibitory p-GSK3β-Ser9 levels [95]. An inhibition of GSK3β-Ser9 phosphorylation by low therapeutic doses of carbon monoxide (CO) enhanced endothelial cell migration and vessel repair in a vascular injury mouse model, a process involving chromatin remodeling [102]. Moreover, in atherosclerosis-prone apolipoprotein E (ApoE) KO mice, increased GSK3β serine phosphorylation (presumably including Ser9) was observed at atherosclerotic lesions [103].

### 4.3. Colitis, Hepatitis, and Peritonitis

#### 4.3.1. Colitis

Oxidative stress, pyroptosis, and inflammation are characteristics of dextran sulfate sodium- (DSS-)induced murine ulcerative colitis (UC). Via the application of the aldehyde dehydrogenase inhibitor disulfiram, total GSK3β protein levels were down, whereas (presumably inhibitory) GSK3β phosphorylation was upregulated in the intestinal mucosa. These effects were accompanied by a significant amelioration of colitis as reflected, amongst others, by reduced levels of malondialdehyde (MDA), cleaved caspase 1, and pro-inflammatory cytokines (TNF, IL-1β, IL-18) as well as Nrf2 upregulation [104] (Figure 3). In other DSS-dependent UC models, colitis was similarly attenuated under conditions involving GSK3β inactivation, e.g., in GSK3 inhibitor- (LiCl [105,106], SB216763 [106], 6-(methylsulfinyl)hexyl isothiocyanate [107]), P2Y_2_ receptor agonist- [108], and CO-treated [105] or CD97 transgenic mice [109]. Equivalent results have been obtained in a murine trinitrobenzene sulfonic acid (TNBS)-induced colitis model, in which GSK3 was inhibited by TDZD-8 [110] (Figure 3).

However, single studies reported increased GSK3β activity in colitis-limiting approaches, e.g., increased p-GSK3β-Tyr216 levels following injection of the peroxisome proliferator-activated receptor (PPAR-)γ modulator GED-0507-34 Levo [111]. Interestingly, murine TNBS–induced colitis is prone to spontaneous resolution of chronic inflammation towards a significantly milder inflammatory status in combination with sustained fibrosis. This process involves IL-13-induced inactivation of GSK3β via Ser389 phosphorylation in lamina propria mononuclear cells and a subsequent decrease in pro- (IL-23, -17) and increase in anti-inflammatory cytokines (IL-10) [112].

#### 4.3.2. Hepatitis

In mice suffering from carbon tetrachloride (CCl_4_)-induced hepatitis, disease severity—comprising hepatic injury, steatosis, and elevated serum levels of alanine (ALT) and aspartate aminotransferases (AST)—could be significantly attenuated, when the animals were pre-treated with methylene blue, a substance that concomitantly reduced the net activity of GSK3β in the liver [113]. Following the establishment of fulminant hepatitis via concanavalin A injection in both WT and protein tyrosine phosphatase receptor type O KO mice, the KO showed enhanced GSK3β-Ser9 phosphorylation together with reduced indication of inflammation (reduced TNF, IL-1β, Il-6, IFN-γ, CCL2, 3, and CXCL10 levels; reduced degree of immune cell infiltration) [114] (Figure 3. Moreover, in murine models of acute liver failure (induced by the combination of D-galactosamine and LPS), pretreatment with SB216763 led to considerable reduction of lethality [115], liver injury, ALT/AST serum levels, and hepatic TNF, IL-1 β, and IL-6 [115,116] as well as CCL-1 and -2, CXCL-1 and -10 expression (Figure 3). This process involved the GSK3β inhibition-dependent activation of autophagy [116]. Using the same experimental models, an equivalent improvement could be achieved under alternative conditions linked to GSK3β inhibition, e.g., application of N-acetylcysteine [117], sodium phenylbutyrate, or overexpression of stress-associated endoplasmic reticulum protein 1 [118]. Similarly, induction of GSK3β-Ser9 phosphorylation by curcumin and/or ascorbic acid in mice [119] or by L-carnitine in rats [120] coincided with the prevention of lead-induced liver-injury. In detail, parameters such as ALT/AST levels, tissue damage, oxidative stress markers (e.g., increased MDA, reduced glutathione (GSH) and superoxide dismutase (SOD) levels), and inflammation (e.g., hepatic TNF and NF-κB-p65 levels) were significantly ameliorated [119]. Equivalent effects have been described in *Burkholderia pseudomallei*-infected mice, in which survival, bacterial burden in the liver, and serum cytokine levels (increase in TNF, IL-1β, IL-18, and IFN-γ; decrease in IL-4 and Il-10) were improved by chloroquine, a treatment also leading to inactivation of liver GSK3β via p-Ser9 [121]. It should also be noted that in a high-fat diet-induced model of fatty liver hemorrhagic syndrome in laying hens, which is, amongst others, characterized by increased hepatic oxidative stress and inflammation (TNF, IL-1β, -6, and -8 mRNA), an amelioration could be achieved using salidroside. For this herbal drug, a suppression of activating GSK3β-Tyr216 phosphorylation was demonstrated in primary chicken hepatocytes [122].

#### 4.3.3. Peritonitis

In murine Pam3CSK4-induced peritonitis, the number of inflammatory cells in the peritoneum was significantly reduced in GSK3β^+/−^ mice or in the absence of one or both GSK3β alleles in bone marrow cells. In WT animals, the application LiCl yielded comparable results [67]. For another substance leading to GSK3β-inhibition, i.e., ephedrine hydrochloride, anti-inflammatory effects have been shown in the murine peptidoglycan-induced peritonitis model, as reflected by increased IL-10 and decreased TNF, IL-1β, and IL-6 secretion by peritoneal macrophages [123].

### 4.4. Diabetes 

**Figure 4:**
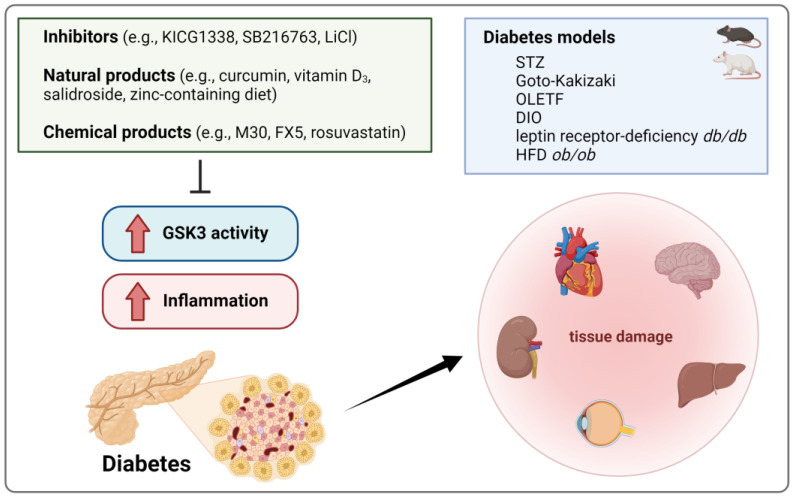
Increased GSK3 activity in diabetes is associated with enhanced inflammation and tissue damage in several organs. Inflammatory and destructive features of diabetes can be alleviated by GSK3 inhibition in different diabetes models, indicating a functional connection between GSK3 and diabetic alterations. STZ, streptozotocin; OLETF, Otsuka Long-Evans Tokushima Fatty rats; DIO, diet-induced obese; HFD, high-fat diet. Created with BioRender.com.

Murine experimental diabetes, commonly induced by streptozotocin (STZ; a glucosamine nitrosourea with strong β cell cytotoxicity), includes various characteristics of inflammation [124]. Thus, significant grades of inflammation and tissue damage have been described in the hearts [125], livers [126], kidneys [127], eyes [128], and brains [129,130] of mice with STZ-induced hyperglycemia (Figure 4). This included elevated IL-1β, IL-6 [129], CCL2 and 5 [128], TNF, and plasminogen activator inhibitor 1 (PAI-1) levels, lipid and 3-nitrotyrosine accumulation [125,126,127], immune cell infiltration [128], cardiac [125] and renal fibrosis [127], hippocampal neurodegeneration [130], as well as hepatic necrosis, apoptosis, and damage-associated inflamed foci [126]. Though single studies reported the opposite [131], these events were generally connected to increased GSK3β levels [129,130] and/or enhanced GSK3β activity as represented by reduced Ser9 phosphorylation [125,126,127,130] and could be prevented, or at least confined, by GSK3 inhibition (SB216763) [125,132]. A comparable amelioration of inflammation was observed in various other rodent diabetes models, e.g., diet-induced obese, leptin receptor-deficient *db/db* [133], and HFD *ob/ob* transgenic mice [134] or STZ-treated diabetic [135], Goto-Kakizaki [136], and Otsuka Long-Evans Tokushima Fatty rats [133] following the application of GSK3 inhibitors (e.g., KICG1338 [133], LiCl [136]). Alternatively, GSK3β-Ser9 phosphorylation-inducing natural (e.g., curcumin [135], vitamin D_3_ [137], salidroside [138], zinc-containing diet [127]) and chemical products (e.g., iron chelator M30 [134], glucocorticoid receptor antagonist FX5 [139], rosuvastatin [137]) have been used (Figure 4).

### 4.5. Neuroinflammation

As the literature reporting evidence for the connection between GSK3 and neuroinflammation is too extensive to be fully considered here, we will focus in the following on the involvement of GSK3 in neuropsychiatric and neurodegenerative disorders (even if these are not easy to separate), using selected examples of depression, AD, and PD. In addition, ischemic stroke will be included in our evaluation.

#### 4.5.1. Neuropsychiatric Diseases

**Figure 5:**
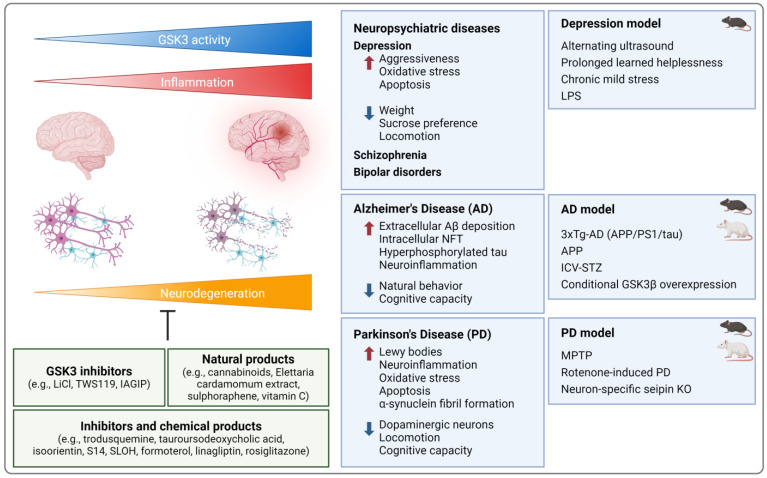
Increased GSK3 activity in neuropsychiatric (depression, schizophrenia, bipolar disorders) and neurodegenerative diseases (AD, PD). In different disease models, GSK3 inhibitors, natural, and chemical products reduce disease symptoms and inhibit GSK3 activity. AD, Alzheimer’s disease; NFT, neurofibrillary tangles; PD, Parkinson’s disease; LPS, lipopolysaccharide; 3xTg, APP/PS1/tau triple-transgenic; APP, amyloid precursor protein; PS1, presenilin 1; ICV-STZ, intracerebroventricular streptozotocin injection; MPTP, 1-methyl-4-phenyl-1,2,3,6-tetrahydropyridine-induced PD. Created with BioRender.com.

Due to its contribution to the shape of the neuronal architecture of the hippocampus, e.g., via adjusting neurogenesis, neurite growth, and synaptic plasticity, GSK3 is also involved in cognitive functions and the broad spectrum of neuropsychiatric diseases [140]. A depression-like phenotype can be induced, for instance, in murine models of learned helplessness [141] and unpredictable alternating frequencies of ultrasound [142] as well as rat models of chronic (unpredictable) mild stress [143,144,145] (Figure 5). The respective rodents showed enhanced aggressiveness [142], but reduced weight gain [143,144,145], sucrose preference, and locomotion [142,143,144]. In the hippocampus, increased oxidative stress (higher MDA, lower SOD levels), apoptosis (e.g., elevated caspase-3), and inflammation were detected (activated NF-κB, increased caspase-1, and NLRP3 [143] as well as TNF, IL17A, IL-23 [141], IL-1β, and IL-6 levels in the hippocampus; microglia activation; elevated plasma TNF levels [142]). These symptoms were accompanied by elevated hippocampal GSK3α/β mRNA and protein levels [142] as well as reduced GSK3α-Ser21 [141] and GSK3β-Ser9 phosphorylation in the hippocampus [141,143,144] and the prefrontal cortex [145]. In the rat model, LiCl [145], fluoxetine, the polyphenol baicalin [143], and the flavonoid dihydromyricetin [144] improved behavioral and physiological indications of depression and restricted GSK3β activation (Figure 5).

Since neuroinflammation is strongly implicated in the development of depression-like behavior, the creation of pro-inflammatory conditions in the brain is suitable to generate murine models of depression [146]. In response to LPS, for instance, mice developed neuroinflammation and oxidative stress (higher MDA levels, microglia activation, increased hippocampal p65, IL-1β [147], NLRP3, caspase-1, IL-18 [148], TNF, and IL-6 levels [144]) and displayed behavioral alterations as described for the aforementioned models [144,147,148]. Again, increased GSK3β protein expression [148] and activation due to reduced p-GSK3β-Ser9 levels [147] defined this disease model (Figure 5). Recovery, at least in part, could be achieved using (low-dose) esketamine [148], dihydromyricetin [144], or the combination of escitalopram and doxycycline [147]. Deficiency for specific genes, e.g., the calcium-activated potassium channel KCa3.1, also improved LPS-induced neurologic health issues in KO mice, while enhancing GSK3β-Ser9 phosphorylation [149]. Roughly comparable observations concerning the impact of GSK3 expression/activation on neuropsychiatric conditions have been made in schizophrenia and bipolar disorder [150].

#### 4.5.2. Neurodegenerative Diseases

Though the primary cause of neurodegenerative AD is still unknown and remains to be elucidated, significant characteristics are the extracellular deposition of amyloid-β (Aβ) peptides, intracellular neurofibrillary tangles (NFT) consisting of hyperphosphorylated tau proteins, and neuroinflammation [151]. GSK3β appears to crucially contribute to most of these aspects [152]. For modeling human tauopathy (including NFT formation, neuro-degeneration, and inflammation [153]), transgenic mice overexpressing disease-causing human tau variants (esp. P301S) have been generated [154] (Figure 5). In the respective animals, tau pathology was associated with activated GSK3β in the brain, as reflected by elevated total GSK3β protein [155] and p-GSK3β-Tyr216 levels [156], decreased p-GSK3β-Ser9 amounts [157], and increased enzymatic activity [158]. Various conditions resulted in both the inactivation of cerebral GSK3β and amelioration of tau-driven disease. In this context, administration of the PPARγ agonist bezafibrate [156] or the third-generation sulfonylurea glimepiride [155], overexpression of TREM2 (triggering receptor expressed on myeloid cells 2) [158], and pharmacological or genetic inactivation of purinergic receptor P2X7 [157] were applied. Beneficial effects include reduced behavioral/cognitive impairment, tau phosphorylation [155,156,157], neuroinflammation (NF-κB levels [155], microglia activation [156,157], COX-2 and inducible NO synthase (iNOS) mRNA/protein levels [156], TNF, IL-1 β, Il-6 mRNA levels [158]), oxidative stress (e.g., GSH levels) [156], as well as neuronal and synaptic loss [158].

Transgenic mice carrying mutated versions of the amyloid precursor protein (APP), either alone [159,160,161] or in combination with presenilin 1 (PS1) [162,163,164] and tau [165,166,167], are also widely used AD models (Figure 5). In some approaches, comorbidities (which may further promote AD development) are mimicked by additives like bacterial LPS [161,165] or viral infection [165]. In general, amyloid pathology, cognitive/behavioral impairment, and neuroinflammation can be observed in combination with markedly increased cerebral GSK3β activation. Accordingly, treatment approaches that improved AD-dependent disorders also reestablished GSK3β-Ser9 phosphorylation in the brain [152]. In APP AD models, for instance, the administration of cannabinoids [159] or PTP1B inhibitor trodusquemine [160] led to GSK3β-Ser9 re-phosphorylation together with improved memory and reduced inflammation (TNF mRNA and COX-2 protein levels [159], microglia activation [159,160]), Aβ levels [159], and neurodegeneration [160]. In APP/PS1 double-mutant mice, treatment with the bile acid tauroursodeoxycholic acid [162], the 6-C-glycosylflavone isoorientin [163], or the phosphodiesterase 7 inhibitor S14 [164] restored GSK3β-Ser9 phosphorylation, attenuated Aβ accumulation in the brain, and reduced tau phosphorylation [162,163,164]. In brain tissue, TNF [162,163], IL1β, and IL-6 mRNA as well as COX-2 protein levels were also reduced [163]. Equivalent improvements have been reported in triple transgenic mice in which treatment with the Aβ-binding carbazole-based molecule SLOH [166] or treadmill exercise [167] resulted in increased p-GSK3β-Ser9 and decreased p-GSK3β-Tyr216 levels.

GSK3β overactivation has also been described in a metabolic model for sporadic AD induced by intracerebroventricular (ICV) injection of STZ. Indication of ICV-STZ-induced AD in rodents (mostly rats) comprises several alterations in the brain, including metabolic, neurochemical, cognitive, and behavioral disturbances, increased levels of Aβ and hyperphosphorylated tau, oxidative stress, as well as neuroinflammation [168] (Figure 5). In such rat models, memory was improved and apoptosis, oxidative stress, inflammation (esp. enhanced TNF, IL-1β, and IL-6), and increased GSK3β (and α [169]) protein levels in the brain were attenuated using the β2 adrenoceptor agonist formoterol [169] or Elettaria cardamomum extract [170]. Similar neuroprotective and anti-inflammatory effects could be achieved using the dipeptidyl peptidase-4 inhibitor linagliptin [171] or the isothiocyanate sulphoraphene [172]. In the latter case, beneficial effects were associated with increased p-GSK3β-Ser9 levels [172]. Contradictory, in another ICV-STZ rat model, AD-like abnormalities were associated with increased GSK3α/β-Ser21/9 phosphorylation, while disease amelioration following the intranasal application of insulin (indicated by improved learning and memory, reduced tau phosphorylation, and microglia activation) occurred in the presence of increased GSK3α/β activity [173]. An AD-like phenotype could also be established in mice by conditional WT GSK3β overexpression in neurons, resulting in typical AD-associated neuronal alterations and severe brain inflammation (apoptotic cells, activated microglia expressing increased levels of TNF, IL-1, IFN-γ, KC, and macrophage inflammatory proteins 1a and 3a) [174]. Further, in several models, pharmacological GSK3 inhibition, e.g., using TWS119 [161] or lithium [165], had positive effects on neuroinflammation.

PD, another progressive neurodegenerative disorder, is mainly characterized by loss of dopaminergic neurons and the occurrence of neuronal inclusion bodies (Lewy bodies), resulting, amongst others, in motor and cognitive deficits. Neuroinflammation is a fundamental driver of PD pathology [175], and GSK3β has been identified as critically involved in the regulation of PD-related cerebral inflammation, oxidative stress [176], and the expression of pathogenic proteins (e.g., α-synuclein) [177]. Rats suffering from rotenone-induced PD, for instance, exhibited disturbances in motor performance and coordination (Figure 5). In the corpus striatum, elevated markers for inflammation (NF-κB, TNF, and IL-1β protein levels) and apoptosis (caspase-3, cytochrome C levels) were measured. Concomitantly, rotenone (an isoflavonoid potently inhibiting mitochondrial complex I electron transport processes [178]) significantly reduced GSK3β-Ser9 phosphorylation, while pretreatment with the 2-oxo-quinoline derivative cilostazol strongly increased inhibitory GSK3β phosphorylation and improved coordinated locomotion, inflammation, and apoptosis [179]. Age-related PD-like deficits can also be induced by neuron-specific seipin KO as reflected by impaired motor coordination, α-synuclein fibril formation in dopaminergic neurons and their progressive decline, as well as increased p-GSK3β-Tyr216 and decreased p-GSK3β-Ser9 levels in aging mice [180] (Figure 5). Moreover, these mice were prone to enhanced neuroinflammation, either age-related (IL-6 protein levels) [180] or under pro-inflammatory conditions, e.g., following intracerebroventricular Aβ injection (hippocampal TNF and Il-6 protein levels, microglia and astrocyte activation) [181]. Application of the PPARγ agonist rosiglitazone alleviated α-synuclein oligomerization and neuronal loss, improved locomotion [180] and inflammation, and normalized GSK3β activity [180,181].

Experimental PD was also established in various animals using the neurotoxic meperidine analog MPTP (1-methyl-4-phenyl-1,2,3,6-tetrahydropyridine; Figure 5). PD-mimicking symptoms resulting from (predominantly intraperitoneal) MPTP injection include neurotoxicity and neuronal apoptosis, oxidative stress, α-synuclein oligomer formation, and neuroinflammation [182]. In this model, motor deficits, loss of dopaminergic neurons [183,184], and an increase in inflammatory markers (e.g., activated astrocytes; increase in IL-1β [183,184], TNF, IL-6, CCL2 and 3 [183], IL-18, and COX-2 mRNA [184]) could be observed. These symptoms could be prevented by the intravenous injection of the IKK-activated GSK3β inhibitory peptide [183] (IAGIP; a peptide-binding and inhibiting GSK3β following its sequential phosphorylation by IKK and GSK3 [185]). In combined MPTP-treated mouse and cell culture experiments, the connection among anti-inflammatory/neuroprotective effects and reduced GSK3β activation has also been shown using vitamin C [186]. In contrast, in mixed primary glial cells, an increase in GSK3β-Ser9 phosphorylation under neuroinflammatory conditions (i.e., treatment with neurotoxic 1-methyl-4-phenylpyridinium) was observed, while p-GSK3β-Ser9 levels were reduced in glia cells deficient for NLRC5 (nucleotide-binding oligomerization domain-like receptor family caspase recruitment domain containing 5), though the latter represents a condition that improves MPTP-induced PD in mice [184].

#### 4.5.3. Ischemic Stroke

In rats [187,188,189,190,191] and mice [192,193,194,195,196,197,198,199,200], ischemic stroke can be provoked by transient or permanent middle cerebral artery occlusion (MCAO) [201] (Figure 3). Mortality [194,197], neurological deficits, infarct volume, brain edema [187,192], oxidative stress (increased ROS and MDA [189], reduced SOD activity/GSH levels [200]), apoptosis (e.g., caspase-3 [187,192] and -9 activation [189]), and inflammation (number of astrocytes, monocytes/macrophages, and infiltrating neutrophils [187]; iNOS, TNF, IL-1β, MCP-1, and p-p65 levels [192]) were reduced when GSK3 activity was suppressed (Figure 3). This could be achieved by GSK3 inhibition (inhibitor VIII [187], SB216763 [188,195]) or by a large variety of p-GSK3β-Ser9 elevating substances. The latter include curcumin [202], salvianolic acid A [194], peptide hormone apelin 13 [189], the alkaloid evodiamine [196], medicarpin (a pterocarpan-type phytoalexin) [197], the naphthoquinone 2-methoxystypandrone [198], ginkgolide K [199], or lipid emulsion [190]. For large molecules with restricted blood–brain barrier permeability, the generation of smaller functional variants may improve their applicability, as described for a peptide fragment of adiponectin comprising its globular domain [200].

Beyond pharmacological intervention, the allocation of an enriched, stimulatory environment may also contribute to re-increased GSK3β-Ser9 phosphorylation in the affected animals [191]. In addition, transplantation of human umbilical cord-derived mesenchymal stem cells, especially in combination with curcumin, enhanced p-GSK3β-Ser9 levels and significantly reduced the extent of brain damage and apoptosis, neurological deficits, oxidative stress, and neuroinflammation in MCAO-induced acute ischemic stroke [202]. An influence of the genetic background, in which the respective model was applied, has also been reported. While deletion of the extra-cellular matrix (ECM) molecule mindin involved enhanced p-GSK3β-Ser9 levels together with an improved outcome following ischemic stroke in mice [192], absence of E3 ubiquitin ligase RING finger protein (RNF) 8 had the opposite effect. In comparison to the WT MCAO model, this was reflected by the combination of further decreased p-GSK3β-Ser9 levels and increased neuronal injury, apoptosis, oxidative stress, and inflammation in RNF8 KO mice. Application of the ECM protein reelin (a factor supporting neuronal development [203]), however, normalized inhibitory GSK3β phosphorylation and revoked the RNF8 KO-dependent aggravation of MCAO-induced defects [204]. In murine neonatal hypoxic–ischemic brain injury models, the application of GSK3 inhibitors tideglusib [205] or SB216763 [206] also reduced brain infarction [205] and mediated neuroprotective effects by reducing oxidative stress (e.g., by increasing SOD), neuroinflammation (increased IL-10; decreased TNF and IL-6), and neuronal apoptosis (reduced caspase-3 activation) [206].

### 4.6. Pulmonary Diseases

Following the induction of acute pneumonia using either influenza A virus (IAV) or LPS, lung tissue alterations and pulmonary inflammation (immune cell invasion, elevation of TNF, IL-1β, -6, and -8 in damaged lung tissue, M1 polarization of macrophages) in mice could be reduced by ABPA1 (Aloe Vera Barbadensis extract C-derived polymeric acemannan; Figure 3). In both LPS-treated RAW264.7-derived macrophages and IAV-infected lung tissue, this substance elevated GSK3β-Ser9 phosphorylation [207]. In another murine LPS-dependent ALI model, a concomitant increase in p-GSK3-Ser9 and the reduction of lung injury, oxidative stress (e.g., GSH, SOD), and inflammation (leukocytes, TNF, IL-1β, and Il-6 in the bronchoalveolar lavage fluid (BALF); iNOS and COX-2 in lung tissue) could be achieved applying the prenylflavonoid xanthohumol [208]. The use of benzothiazepinone compounds (acting as non-ATP-competitive GSK3 inhibitors) [209] and PPAR-β/δ agonist GW0742 in mice [210] or the α2-adrenergic receptor agonist dexmedetomidine in rats [211] yielded comparable results. When LPS-induced lung inflammation was further aggravated by p47^phox^ KO, p-GSK3β-Ser9 levels were lower in KO than in WT macrophages [212]. An alternative (septic) ALI model can be created via the cecal ligation and puncture technique (Figure 3). Here, treatment of the respective rats with mitochondrial coenzyme Q enhanced GSK3β-Ser9 phosphorylation in lung tissue, an effect associated with increased survival, reduced lung injury and edema, and decreased oxidative stress (myelopereoxidase activity) and inflammation (IL-6, KC, and macrophage inflammatory protein 2 levels) in lung tissue [213]. Further, newborn rats exposed to hyperoxic surroundings exhibited lung tissue injury, oxidative stress (e.g., Nrf2 downregulation), and inflammation (immune cells and levels of TNF, IL-1β, and IL-6 in BALF and lung tissue, nuclear p65 accumulation) in combination with GSK3β upregulation in the lung. The treatment with genipin, an aglycone derived from geniposide, resulted in both the downregulation of GSK3β mRNA and the attenuation of the hyperoxia-induced symptoms [214]. Comparably, GSK3β activity was enhanced in alveolar macrophages of rats suffering from *pneumocystis carinii*-induced pneumonia, while healthy animals had markedly higher p-GSK3β-Ser9 levels [215].

Again, in several alternative models, opposing results have been obtained. Major indications of airway inflammation in a murine house dust mite-induced asthma model are enhanced inflammatory cells in peribronchial/perivascular areas and BALF, IgE levels in BALF and serum, and cytokine levels (IL-4, -5, -13, and eotaxin-1) in BALF and lung homogenates. These effects could be prevented in the presence of melatonin, which also decreased p-GSK3β-Ser9 levels that were elevated under pro-inflammatory conditions in airway smooth muscle cells (i.e., following TGF-β stimulation) [216]. In mice with ovalbumin-induced asthma, significantly increased p-GSK3β-Ser9 levels were associated with reduced lung function and pulmonary inflammation, i.e., increased numbers of leukocytes and levels of TNF, IL-2, -4, -5, -13, -33, and IFN-γ in BALF, enhanced serum IgE, inflammatory cell invasion and NF-κB-p65 phosphorylation in lung tissue. *Vice versa*, asthma could be improved under conditions, in which an activation of GSK3β could be observed, such as the application of dexamethasone or Louki Zupa decoction [217]. Moreover, in guinea pigs, pulmonary inflammation (i.e., airway invasion of inflammatory cells) that characterizes LPS-provoked chronic obstructive pulmonary disease (COPD) could not be prevented by SB216763 pretreatment, while signs of tissue remodeling could [218].

Together, these data reflect well the situation in the other diseases described above by suggesting that activated GSK3β is involved in the pathogenesis of inflammation-associated disease, while its inhibition may represent a promising contribution within treatment. The existence of conflicting results nevertheless reminds us that this conclusion does not apply without restriction.

### 4.7. Fibrosis

Fibrosis may occur as a subsequent phenomenon in most (esp. chronic) inflammatory diseases [219]. Mechanistically, fibrosis results from dysregulated or excessive tissue repair processes, e.g., in the course of repetitive or severe tissue injury, and can affect every organ [220]. As GSK3 is strongly involved in processes like ECM formation and epithelial-mesenchymal transition, it has been recognized as a crucial regulator of fibrosis [221]. In the following chapter, the role of GSK3 in fibrosis will be discussed using hepatic and pulmonary fibrosis as examples.

#### 4.7.1. Liver Fibrosis

In a rat model of diethylnitrosamine (DEN)-induced liver fibrosis, reduced body/liver weight, signs of liver inflammation and injury (inflammatory infiltration, steatosis, fibrous septae, collagen accumulation), increased liver enzyme activity (e.g., alkaline phosphatase (ALP), AST, ALT), and hepatic oxidative stress (e.g., increased MDA and lipid oxidation, decreased SOD) were observed. These effects were accompanied by significantly higher GSK3β protein expression levels. The bioflavonoid morin, either protectively or therapeutically applied, significantly ameliorated DEN-dependent liver fibrosis and decreased GSK3β expression [222]. Rats [223] and mice [224,225] with CCl_4_–induced liver cirrhosis/fibrosis were also characterized by severe histological changes (disruption of hepatic tissue architecture [223,224,225], formation of large fibrous septae, collagen accumulation [223]), and enhanced serum levels of fibrosis biomarkers such as ALT, AST [224,225], ALP, bilirubin, hyaluronic acid, and laminin [223]. In the livers of these rodents, reduced p-GSK3β-Ser9 levels were detected [223,224,225], while the infusion of umbilical cord blood-derived mesenchymal stem cells [223] or the application of *Pheretima aspergillum* (i.e, earthworm) extract [225] re-established Ser9 phosphorylation and reduced indication of fibrosis. Fibrosis in combination with significantly reduced p-GSK3β-Ser9 levels was equivalently observed in HFD/STZ-treated diabetic mice, and both effects could be reversed by a polysaccharide extracted from *Abelmoschus esculentus* (L.) Moench (i.e., okra) [226].

Differing results, however, have been reported in NAFLD mouse models, in which GSK3β appears to be predominantly inactive as demonstrated by significantly increased GSK3β phosphorylation levels [227,228]. Vice versa, the application of the tetracyclic triterpenoid actein dose-dependently reduced both p-GSK3β-Ser9 and hepatic fibrosis [228]. Moreover, following bile duct ligation (BDL), cholestatic liver fibrosis (CLF) was aggravated in mice treated with SB216763 [229]. Another study reported that the prevention of CCl_4_–induced liver fibrosis by the iridoid glycoside hastatoside appears to depend on its ability to bind to GSK3β and enhance its activity [230].

#### 4.7.2. Lung Fibrosis

Mice with radiation-induced pulmonary fibrosis (RIPF) were characterized by lower body weight, pneumonitis, hemorrhagic lung tissue, thickened alveolar septae, collagen deposition, fibrotic lesions [231], and higher expression of GSK3β mRNA [231] and protein [232] than controls. Accordingly, overexpression of a GSK3β-targeting miRNA mimic (resembling miR-155-5p) significantly reduced irradiation-induced GSK3β protein expression and mitigated the development of RIPF (reduction of histological changes and collagen accumulation) [232]. Intraperitoneal administration of the GSK3 inhibitor 9ING41 had comparably beneficial effects in mice with *Streptococcus pneumonia*-induced empyema (improved lung volume and function, reduced pleural thickness, and myofibroblast accumulation). Moreover, 9ING41 markedly reduced activated pleural GSK3β (i.e., p-GSK3β-Tyr216) [233].

In contrast, STZ-treated mice suffering from diabetic pulmonary fibrosis exhibited increased levels of p-GSK3β-Ser9 in lung tissue, whereas the significant amelioration of fibrosis by inactivation of the CIP4 (Cdc42-interacting protein-4) gene was accompanied by reduced p-GSK3β-Ser9 amounts [234]. In silica dust-exposed rats with pulmonary fibrosis, reduced expression of total GSK3β protein and increased amounts of GSK3β with negative regulatory phosphorylation [235] could be detected. Transplantation of bone marrow-derived mesenchymal stem/stromal cells (BMSC) or application of BMSC-conditioned medium, however, re-established GSK3β and reduced p-GSK3β-Ser9 levels, a condition also resulting in the attenuation of fibrosis [235].

For other organs (e.g., heart, kidney, or intestine), equivalently versatile results have been described. Therefore, it has to be assumed that GSK3 acts as an ambivalent control module within the development and progression of fibrosis by mediating both pro- and anti-fibrotic effects [221].

**Table 1:** GSK3 inhibitors used in animal models of inflammation and tissue injury.

Inhibitor	Disease	Animal Model	Organ/Tissue Analyzed	References
TDZD-8	Arthritis	CIA (mouse)	Cartilage/bone	[84,86]
		CIA (rat)	Cartilage/bone	[85]
		CFA (mouse)	Cartilage/bone	[90]
	Colitis	TNBS (mouse)	Intestine	[110]
LiCl	Arthritis	CIA (mouse)	Cartilage/bone	[84]
		CAIA (mouse)	Cartilage/bone	[67]
	Cardiovascular	AAA (rat)	Aorta	[101]
		MIRI (rat)	Heart	[95]
	Colitis	DSS-UC (mouse)	Intestine	[105,106]
	Peritonitis	Pam3CSK4 (mouse)	Peritoneum	[67]
	Diabetes	Goto-Kakizaki (rat)	Pancreas	[136]
	Depression	Chronic mild stress (rat)	Brain	[145]
	AD	3xTg-AD (mouse)	Brain	[165]
VA	Arthritis	CIA (mouse)	Cartilage/bone	[86]
SB216763	Colitis	DSS-UC (mouse)	Intestine	[106]
	Hepatitis	ALF (mouse)	Liver	[115,116]
	Diabetes	STZ (mouse)	Heart	[125]
		STZ (mouse)	Brain	[132]
	Ischemic stroke	MCAO (mouse)	Brain	[188]
		MCAO (rat)	Brain	[195]
	HIE	CAL/hypoxia (mouse)	Brain	[205]
	COPD	LPS (guinea pig)	Lung	[218]
	CLF	BDL (mouse)	Liver	[229]
6-MITC	Colitis	DSS-UC (mouse)	Intestine	[107]
KICG1338	Diabetes	STZ (mouse, rat)	Various (e.g., muscle, liver, pancreas)	[133]
TWS119	AD	APP (mouse)	Microglia	[161]
IAGIP	PD	MPTP (mouse)	Brain	[183]
inhibitor VIII	Ischemic stroke	MCAO (mouse)	Brain	[187]
tideglusib	HIE	CAL/hypoxia (mouse)	Brain	[205]
BTZ-6j, -3j	Pulmonary	ALI (mouse)	Lung	[209]
9ING41	Empyema	*S. pneumonia (mouse)*	Lung	[233]

TDZD-8, thiadiazolidinone-8; VA, valproic acid; 6-MITC, 6-(methylsulfinyl)hexyl isothiocyanate; IAGIP, IKK-activated GSK3beta inhibitory peptide; HIE, hypoxic–ischemic encephalopathy; COPD, chronic obstructive pulmonary disease; CLF, cholestatic liver fibrosis; BDL, bile duct ligation; BTZ, benzothiazepinone. Further abbreviations: see previous Figures. Please note that natural/chemical compounds have been only included if direct GSK3-inhibiting capacity was demonstrated.

### 4.8. A Reflection on Contradictory Results

In most cases, the attempt to mold human inflammatory disease in animal models leads to (at least seemingly) contradictory results among different models or (though less frequently) within the same model, and this also applies to the animal studies available for GSK3. Though most studies state an involvement of enzymatically active GSK3(β) in the development and progression of diseases with inflammatory, destructive, and fibrotic components, other reports imply an inactive state under these conditions. As an ordinary explanation, these discrepancies may be ascribed to differences (in our case, in the functional effects of GSK3) among different cell types, tissues, organs, organisms, and strains used [221,236,237]. These specific effects (e.g., the precise integration of GSK3 in the various potential signaling pathways) may not be fully characterized in every case [238]. Other aspects of experimental design can further play a role, such as age of the animals, the model applied (i.e., was a spontaneous, an experimental, or a genetic model used? [236]), disease duration at the time of evaluation, and the extent of analyses (e.g., was the analysis performed in isolated cells, selected tissues, or whole organs?). Environmental factors (ventilation, nutrition, pathogens, etc.) can also massively impair the outcome of studies at different sites (e.g., among different working groups or when laboratories move) [239]. Moreover, it may be of importance whether a selected model is based on direct or indirect induction methods, i.e., whether disease in the organ of interest is provoked by a treatment specifically affecting this organ or by a broader approach such as diabetes, which involves major disturbances throughout several organs. However, in addition to these “usual” explanations, inconsistencies may be predominantly due to the complexity of inflammatory diseases that cannot fully be reflected by, albeit elaborated, disease models, which can only represent parts of the respective pathogenic processes. Thus, they may access a merely limited selection of involved regulatory pathways as well as the multifaceted cellular and molecular aspects that contribute to a particular disease [221]. For AD, for instance, it has been described that several models share common symptoms, while no model exists that represents the entire syndrome [236]. Thus, a specific bias (obviously also applying to GSK3) may be inherent in different models, leading to results perceived as contradictory.

## 5. Conclusions

**Figure 6:**
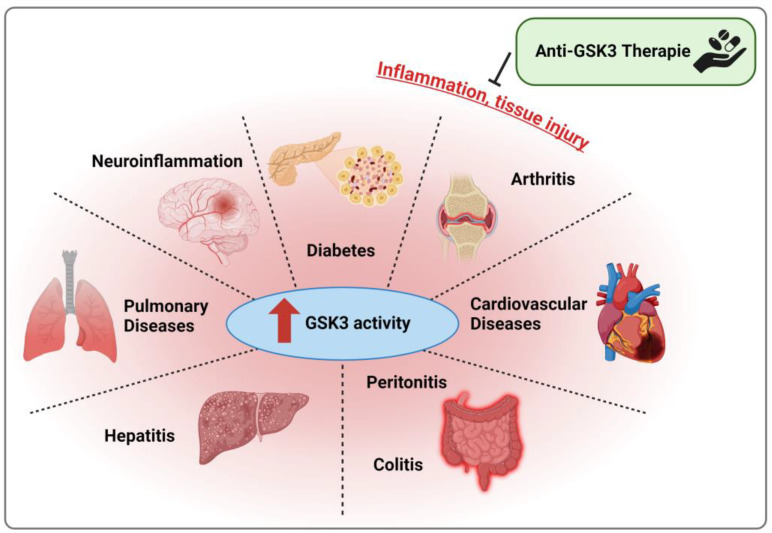
GSK3 activity promotes inflammation-related injury in various tissues and diseases. As GSK3 inhibition mostly improves disease conditions and reduces signs of inflammation, GSK3 seems to be a promising therapeutic target to study for multiple inflammatory diseases. Created with BioRender.com.

The contribution of GSK3 to the initiation and development of inflammation and inflammatory diseases has become more and more evident. Though we have described previously that overall, GSK3 is able to mediate both pro- as well as anti-inflammatory effects (depending on the specific conditions present), and to act as a key regulator balancing progression and resolution of inflammation [2], the message obtained from the animal models summarized in this work is more consistent (despite a few opposing studies). In general, (over-)activated GSK3β, mainly indicated by reduced inhibitory phosphorylation, is strongly involved in intensifying inflammatory and destructive processes resulting in cell death, tissue damage, oxidative stress, and persistent inflammation. *Vice versa*, substances or conditions leading to the recovery of GSK3β-Ser9 phosphorylation (or other forms of its inactivation) result in the amelioration of disease severity, including cell, tissue, and organ protection, functional preservation, and the amelioration of inflammation (Figure 6). To some extent, the janiform nature of GSK3 may still be present, as reflected in the couple of publications reporting deviating results, but within most models, GSK3 appears as a master switch, whose deactivation is strongly connected to improved health conditions. This renders GSK3 a still-promising candidate for pharmacological intervention, and various GSK3 inhibitors are still under clinical investigation, although previous studies could not completely fulfill the high expectations [240]. However, until safe, effective, and specific GSK3-targeting anti-inflammatory therapy can be implemented, much more research is necessary to further elucidate the regulatory impact of GSK3 on inflammation and the molecular mechanisms by which this enzyme governs deleterious events. Certainly, these efforts will require current, but also new approaches in studying animal disease models, which will remain an essential tool to address these open questions.
